# Isolating and Identifying One Strain with Lead-Tolerant Fungus and Preliminary Study on Its Capability of Biosorption to Pb^2+^

**DOI:** 10.3390/biology13121053

**Published:** 2024-12-16

**Authors:** Wanna Li, Liujing Zhao, Cui Liu, Xingpeng Bai, Chenyuan Xu, Fengqiu An, Feilong Sun

**Affiliations:** School of Environmental and Chemical Engineering, Xi’an Polytechnic University, Xi’an 710600, China; liwanna0226@163.com (W.L.); liuliujing2350381344@gmail.com (L.Z.); 19225290507@163.com (C.L.); 17393762199@163.com (X.B.); 15252547893@163.com (C.X.)

**Keywords:** lead, Sarocladium, SEM, XRD, FTIR

## Abstract

To obtain lead-resistant microorganisms as potential strains for bioremediation, in this study, a strain of fungus with high resistance to lead was isolated and domesticated from lead-contaminated soil, which was cultured and molecularly biologically identified as the genus Sarocladium Pb-9 (GenBank No. MK372219). The optimal incubation time of strain Pb-9 was 96 h, the optimal incubation temperature was 25 °C, and the optimal incubation pH was 7. The strain Pb-9 had a good adsorption effect on Pb^2+^ at a lead concentration of 2000 mg/L; scanning electron microscopy (SEM) observed that the spores of the Pb-9 strain appeared to be wrinkled and deformed under Pb^2+^ stress, and XRD analysis showed that the mycelium of Pb-9 adsorbed Pb^2+^; Fourier transform infrared spectroscopy (FTIR) analysis showed that the Pb-9 strain might produce substances such as esters and polysaccharides under the treatment of different Pb^2+^ concentrations. The above results showed that strain Pb-9 has good resistance and adsorption capacity to lead. Therefore, it has potential application value in the bioremediation of environmental heavy metal pollution, and this study provides a fundamental basis for the bioremediation of lead pollution in the environment.

## Simple Summary

In this experiment, a strain of Pb-resistant fungus (No. Pb-9) was isolated and investigated from heavy metal-contaminated soil. The molecular biology method identified the strain (Pb-9) as *Sarocladium*, and scanning electron microscopy, infrared spectroscopy, and X-ray analysis were carried out to compare and analyze the changes in the micro-morphology of the strain and its adsorption of Pb^2+^ in different Pb^2+^ concentration treatments. Strain Pb-9 is expected to be developed and bred as a potential dominant strain in the bioremediation of soil heavy metal pollution.

## 1. Introduction

Heavy metal pollution of soil and water has become an increasingly prominent environmental problem, of which lead is an important pollutant among heavy metals. It mainly comes from minerals, metal smelting, leaded gasoline, municipal sewage, industrial waste, and paint spraying. Lead can remain in the soil for 150 to 5000 years and is ingested by humans through the consumption of crops grown in contaminated areas, causing harmful effects on the human nervous system [1]. Therefore, the long-term presence of these heavy metal pollutants in the environment is bound to exacerbate the threat to human health. To solve the problem of heavy metal contamination in the environment, many conventional types of technologies have been utilized for treatment such as reverse osmosis, ultrafiltration, nanofiltration, ion exchange, flotation, electrodialysis, chemical precipitation, and coagulation. But these methods suffer from the disadvantages of high cost, low removal rates, and generation of secondary contaminants. Many organizations and scientists from different disciplines have indicated that bioremediation can be used to reduce metal availability below the permissible limits [2]. Microorganisms, which have a large specific surface area and strong adsorption activity for heavy metals, are less likely to cause secondary pollution, and the use of microorganisms’ metabolites to react with heavy metals can minimize the impact of the remediation process on the local ecological environment. With the development of biotechnology, the application of microbial remediation in the process of heavy metal pollution treatment is becoming more and more widespread.

A large number of studies have found that a certain number of heavy metal-resistant microbial taxa, mainly bacteria, fungi, actinomycetes, and algae, generally exist in soils that have been subjected to long-term stress by one or more kinds of heavy metals [3,4]. Therefore, it is very important to isolate and screen out strains with strong adsorption ability among these resistant microorganisms, study their mechanism of action with heavy metals, and use these strains for the bioremediation of heavy metal-contaminated soils; thus, they are crucial technical means at present. Fungi are widely available, fast-multiplying, and vigorous, and because their cell walls contain polysaccharides, proteins, and lipids, which have the potential ability to bind heavy metals, they not only survive in wastewater and soil containing high concentrations of heavy metals, but also are often used as inexpensive adsorbents for heavy metals. Sumathi [5] tested the biosorption properties of the fungal isolates identified in the soil samples collected from the Andaman Islands. The results showed that the fungi were more tolerant to higher concentrations of heavy metals and had a strong uptake capacity for heavy metals. Dusengemungu [6] isolated native filamentous fungi *Aspergillus transmontanensis*, *Cladosporium cladosporioides*, and *Geotrichum candidum spp.* from heavy metal-contaminated soils, and these fungi not only thrived in heavy metal-contaminated soils, but also were able to significantly reduce the amount of soil Cu, Co, Zn, Fe, Mn, and Pb.

*Sarocladium* is a widespread fungus in nature. Some of its species are pathogenic to plants, such as the pathogen causing rice sheath rot, *Sarocladium oryzae*, and the pathogen that can cause disease in sorghum and strawberry, *Sarocladium strictum*. *Sarocladium* is also a plant endophyte that produces secondary metabolites with antimicrobial activity, such as helvolic acid and cerulenin. Anjos [7] reported that compact broom fungus (Sarocladium) is an endophytic fungus prevalent in tropical forage herbaceous plants, and these endophytic fungi may have a very important role in the resistance of host plants to the stress of exotic antibiotics and pollutants. Cebekhulu, S. [8] explored indigenous microorganisms isolated from alkaline tailings such as bacteria (*Sphingomonas*, *Novosphingobium*, and *Solirubrobacter*) and fungi (*Alternaria*, *Sarocladium*, and *Aspergillus*). These microorganisms were found to have the ability to leach potentially toxic and rare earth elements. Cortês [9] reported that crude extracts of *Sarocladium oryzae*, the causal agent of rice sheath rot, were effective in the suppression of rice blasts.

Some reports have shown that secondary metabolites of Serratia marcescens have good insecticidal, fungicidal, and resistance to foreign pollutants. Huanying Ge [10] screened a fungal strain (identified as *Sarocladium terrricola*) with significant pyrene degrading ability from sediments near a petrochemical plant. The growth and pyrene degradation characteristics of the fungus were investigated, and the results showed that the fungus degraded pyrene very well, with a degradation rate of 88.97% in 21 days. Ferdos Ganji and others [11] isolated *Pleurotus eryngii* and *Sarocladium sp.* from the soil of the Sarcheshmeh Copper Mine, one of the largest open-pit copper mines in the world, and were able to withstand a concentration of 5500 ppm copper sulfate. However, not many studies have been conducted on the adsorption of heavy metal lead by *Sarocladium*, isolated from heavy metal-contaminated soil.

## 2. Materials and Methods

### 2.1. Strain

The strain was screened and isolated from the experimental field of the National Loess Fertility and Fertilizer Effectiveness Monitoring Base in Wuquan Town, Yangling Demonstration Area, Shaanxi Province (34°17′51″ N, 108°00′48″ E). The experimental soil was supplemented with Pb(NO_3_)_2_ at 350 mg/kg, and the strain was domesticated by adding different concentrations of Pb(NO_3_)_2_ to the potato glucose medium and named Pb-9.

### 2.2. Media and Reagents

The following components were used analytically pure: peeled potatoes, dextrose (CAS NO: 14431-43-7, purity ≥ 99.5%), magnesium sulfate (CAS NO: 7487-88-9, purity ≥ 99.0%), potassium dihydrogen phosphate (CAS NO: 7778-77-0, purity ≥ 99.5%), peptone (CAS NO: 68308-36-1, purity ≥ 99.0%), agar (CAS NO: 9002-18-0, purity ≥ 99.0%), formaldehyde solution (CAS NO: 50-00-0, purity 36.5–38% in H_2_O), lead nitrate (CAS NO: 10099-74-8, purity ≥99.0%), and potassium bromide (CAS NO: 7758-02-3. purity ≥ 99.0%).

### 2.3. Main Instruments and Equipment

The instruments and equipment employed in this study are as follows: Biochemical Incubator (SPX-250B-Z, Shanghai Boxun Industrial Co., Ltd. Medical Equipment Factory, Shanghai, China); Oscillating Incubator (SHA-C, Changzhou De’ou Instrument Manufacturing Co., Ltd., Changzhou, China); Autoclave Sterilization Kettle (GI54TW, Shanghai Boxun Industrial Co., Ltd. Medical Equipment Factory, Shanghai, China); Optical Biomicroscope (CX41, Olympus Corporation, Tokyo, Japan), lens parameters (eyepiece magnification: 10×, objective magnification: 100×, field of view: 20, numerical aperture: 1.25), bright field, method of observation: transmission; Fourier Infrared Spectroscopy Analyzer (NICOLET5700, Thermo Fisher, Waltham, MA, USA), using the KBr pressing method, DTGS detector, resolution: 4 cm^−1^; Scanning Electron Microscope (FlexSEM1000, Hitachi, Ltd., Tokyo, Japan), accelerating voltage: 20,000 volts, electron beam current: 70,000 nA, reduction of charge accumulation by sputtering a gold film on the surface of a nonconducting sample; X-ray Diffractometer (MiniFlex 600, Rigaku, Ltd., Tokyo, Japan); Freeze-drying Machine (Freeze dryer (FD-1D-50+, BoMedicom Beijing Instrument Co., Ltd., Beijing, China)).

### 2.4. Morphological Observation of the Strain

The strains in the preserved state were inserted into the potato dextrose agar medium (PDA medium) for culture, and the vigorous strains were obtained by expanding the culture step by step. Strain Pb-9 was cultured in an incubator at 25 °C for 3–5 d by the insertion method, and then the mycelium and spore morphology of the active strains were observed by optical microscope.

### 2.5. Molecular Biological Characterization of Strain Pb-9

Fungal amplification of ITS rRNA gene, fungal primers were ITS1: TCCGTAGGTGAACCTGCGG; ITS4: TCCTCCGCTTATTGATATATGC; PCR system (50 μL): 10× LA TaqBufferII (Mg^2+^Plus): 5 μL, dNTP: 8 μL, primer: 1 μL, TaKaRa LA Taq (5 U/μL): 1 μL, DNA template: 2 μL. The PCR system was supplemented with ddH_2_O to 50 μL. The PCR program was as follows: pre-denaturation at 94 °C for 5 min, denaturation at 94 °C for 45 s, annealing at 58 °C for 45 s, extension at 72 °C for 1 minute and 30 seconds, and 31 cycles; the PCR system was stored at 4 °C (preferably for more than 12 h) for 31 cycles. The PCR amplification was detected by electrophoresis, and the PCR products with good amplification effect were sent to Sangon Bioengineering (Shanghai, China). The sequences were compared with the existing sequences in the NCBI database by BLAST to analyze the homology of the strains to be tested, and the phylogenetic tree was constructed by using MEGA 5.0 software.

### 2.6. Determination of the Growth Curve of Strain Pb-9

Using a pipette gun, 1 mL of strain Pb-9 seed solution was inoculated into 100 mL of potato glucose broth medium (PDB medium) and incubated at 28 °C and 130 r/min under constant temperature oscillation for 7 d. Samples were taken every 12 h. The culture solution to be tested was put into centrifugation tubes and centrifuged at 7000 r/min for 5 min, and then the bacterium was put into the oven to bake until it reached a constant weight, and the dry weight of the strain was weighed. The growth curve of Pb-9 was plotted with culture time as the horizontal coordinate and strain dry weight as the vertical coordinate.

### 2.7. Optimization of Culture Conditions for Strain Pb-9

To study the effects of different temperatures and pH values on the growth of Pb-9 under other unchanged cultivation conditions, Pb-9 was placed at 20 °C, 25 °C, 30 °C, 35 °C, and 40 °C, respectively, for cultivation, and the pH values of the medium were adjusted to 4.0, 5.0, 6.0, 7.0, and 8.0 with 1 mol/L NaOH and HCl solutions, respectively. Each treatment was repeated three times, and the growth was characterized by the dry weight size of the strains. After 7 d of incubation with oscillation at 130 r/min, the dry weight of the strains was weighed, and the growth was characterized by the size of the dry weight of the strains.

### 2.8. Studies on the Adsorption Rate of Pb^2+^ by Strain Pb-9

Single colonies of strain Pb-9 were picked and cultured in a PDB medium at 130 r/min and 25 °C for 7 d. After centrifugation at 7000 r/min for 5 min, the supernatant was discarded and the bacterial body was collected, washed with sterile water 2~3 times, and then weighed after dipping the bacterial body into dryness with filter paper to be used as the strain for adsorption.

Pb(NO_3_)_2_ solution was added into 50 mL of PDB medium, so that the lead concentration in the medium was 500 mg/L, 1000 mg/L, 1500 mg/L, and 2000 mg/L, respectively, and 1 g of bacterium was added into each vial, and the strain was fully broken up with a glass rod to mix with the medium, and then cultured with constant temperature oscillation at 24 °C and 130 r/min for 4 d. The bacterium was adsorbed in the same way as the Pb^2+^ concentration (Pb^2+^). A liquid medium with the same Pb^2+^ concentration (without bacteria) was used as the control, and three replicates were set up. At the end of the incubation, the resulting bacterial solution was centrifuged, and the Pb^2+^ concentration was determined using inductively coupled plasma mass spectrometry (ICP-MS) after taking the supernatant. The Pb^2+^ adsorption rate was calculated according to Formula (1).

Q = (C_0_ − C_t_)/C_0_ × 100% (1)

where Q is the adsorption rate of metal ions by the strain, C_0_ is the initial Pb^2+^ concentration (mg/L) before solution adsorption, and C_t_ is the Pb^2+^ concentration (mg/L) in the solution after adsorption.

### 2.9. Scanning Electron Microscopy (SEM) Analysis

Scanning electron microscopy was used to analyze the morphological characteristics of spores of strain Pb-9 under different Pb^2+^ concentration growth conditions. The final concentrations of Pb(NO_3_)_2_ in the PDA medium were 0 mg/L (CK), 2000 mg/L, 3000 mg/L, and 4000 mg/L. Strain Pb-9 was inoculated in the above PDA medium with different concentrations of Pb^2+^, incubated at 25 °C for 5 d, and then removed. The area of vigorous colony growth was selected, and the corresponding area (containing culture medium) was cut into a size of 1 cm × 1 cm and irradiated under UV light for 30 min, then placed in a sealed box and fumigated with formaldehyde vapor for 24 h, and then freeze-dried for 4 h with a vacuum freeze-dryer. The culture medium was removed, and the mycelium was picked and placed on the sample stage of the scanning electron microscope, metal coated, observed, and photographed under the scanning electron microscope.

### 2.10. X-Ray Diffraction (XRD) Analysis

To comparatively analyze whether the Pb-9 bacterium has any adsorption effect on Pb, strain Pb-9 was added into the PDB medium containing Pb(NO_3_)_2_ at a concentration of 4000 mg/L and CK (0 mg/L), respectively, and three replicates were set for each concentration; it was placed in the oscillator at 25 °C and 120 r/min for 5 d and then taken out, and centrifuged for 15 min at 6000 r/min. Bacteria were precipitated, the supernatant was discarded, and the precipitate was rinsed with deionized water, and then centrifuged at 6000 r/min for 15 min, which was repeated twice, and then the precipitate was taken out and dried at 65 °C, and then ground, and analyzed using an X-ray diffractometer. The current of the phototube was adjusted to 15 mA and the voltage was adjusted to 40 KV. The width of the slit was 0.02 deg, the scanning angle speed was 0.25 °/min, the range of the scanning angle was 3°–90°, the average value was taken as the result of the measurement, and the data were analyzed and plotted with the software MDI Jade 6.5 and Origin 8.5.

### 2.11. Infrared Analysis

Strain Pb-9 was inserted into PDB medium containing Pb^2+^ at a concentration of 0 mg/L (CK: blank control) and 4000 mg/L, respectively, and placed in a thermostatic shaker at 25 °C and 150 r/min for 5 d. The strain was removed, and then centrifuged by high-speed cryo-centrifuge at 6000 r/min for 15 min at 4 °C, and then the supernatant was discarded and the bacterial body was collected. The precipitate was lyophilized in a lyophilizer and ground for infrared spectroscopy analysis.

## 3. Results

### 3.1. Morphology and Microstructure of the Strain

**Figure 1:**
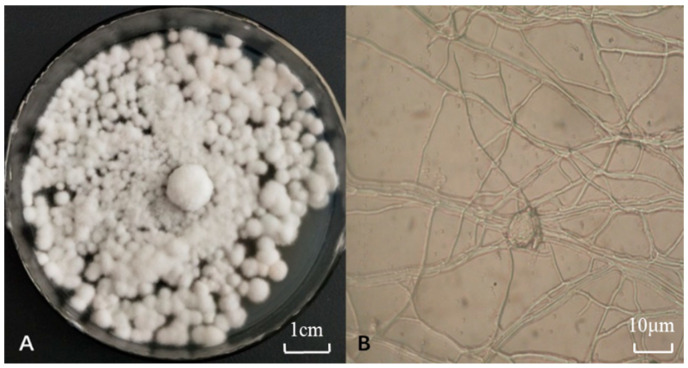
Colony morphology, the microstructure of mycelia, and spores of strain Pb-9 (25 °C, 5 d). Note: (**A**): Pb-9 strain; (**B**): Mycelia and spores (1000×; where the eyepiece is 10×).

As shown in Figure 1, the colony color of strain Pb-9 was white; the aerial mycelium growing on the PDA plate medium showed a white color, as did the mycelium at the base. The mycelium grew denser, and the mycelial villi were longer and grew vigorously (Figure 1A); a large number of mycelium and spores were visible under the microscope (Figure 1B). The mycelium was fine, colorless, full, and rounded, and the spores were smaller in morphology, transparent, and pike-like, scattered around the mycelium. There were obvious sporocarps, which were ovoid, and a large number of spores could be seen gathered in the sporocarps.

### 3.2. Molecular Biology Identification

**Figure 2:**
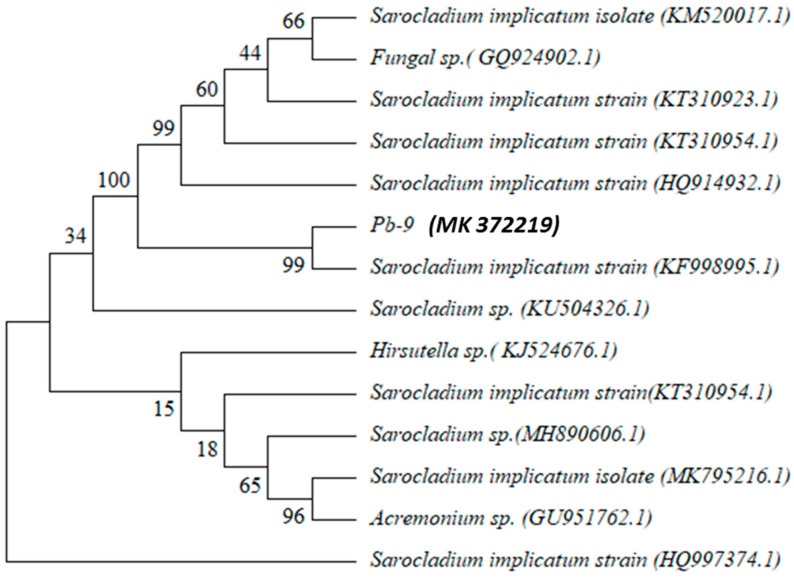
Phylogenetic tree of strains Pb-9.

After PCR amplification, the length of the rRNA gene of strain Pb-9 was about 600 bp, the measured ITS rRNA sequence was compared with the sequence of NCBI by BLAST, and the phylogenetic tree was constructed (Figure 2), which showed that strain Pb-9 was closer to the *Sarocladium implicatum strain*. Therefore, strain Pb-9 was identified as *Sarocladium* with the registration number MK372219 in combination with the morphological characteristics of the strain and the comparison results.

### 3.3. Growth Curve of Strain Pb-9

**Figure 3:**
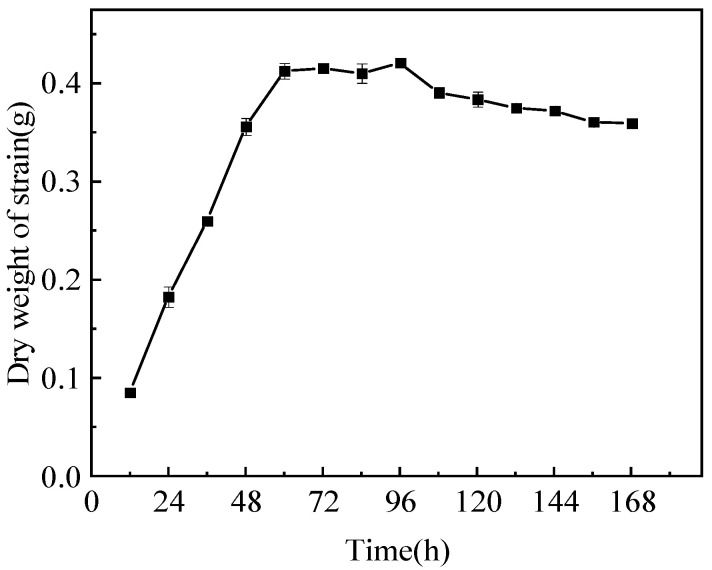
Growth curves of strain Pb-9.

The growth curve of strain Pb-9 is shown in Figure 3. From the figure, it can be seen that Pb-9 was in the rapid growth stage from 0 to 60 h; after 60 h, the bacterium entered the stabilization period, and the dry weight of the strain reached its peak at 96 h, thus choosing the incubation time of 96 h for the subsequent experiments.

### 3.4. Effect of Temperature and pH on the Growth of Pb-9

**Figure 4:**
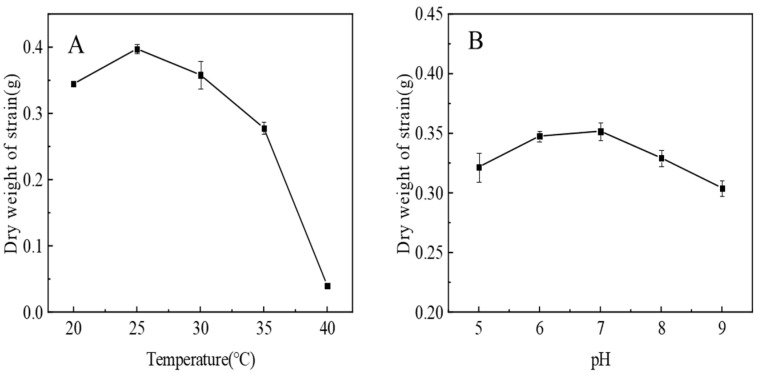
Effect of temperature (**A**) and pH (**B**) on the growth of strain Pb-9. Note: The standard error takes into account the error of the scale.

As shown in the left image of Figure 4A, the dry weight of the Pb-9 strain showed a trend of increasing and then decreasing with the increase in temperature, and the dry weight of the strain reached its highest under 25 °C, which indicated that the optimum culture temperature of strain Pb-9 was 25 °C. Pb-9 could hardly grow at 36 °C, which might be the result of high temperature leading to the death of some strains, or it might be that high temperature negatively affected the metabolic enzymes in the strain and inhibited its growth [12].

Notably, pH can affect the permeability of the cell membrane, the activity of functional groups, the solubility or ionization of intracellular substances, etc. For different strains, their optimal pH ranges are different, and understanding the appropriate pH ranges of strains can help microorganisms be used in production [13]. As shown in the right image of Figure 4B, strain Pb-9 can grow in the range of pH 5~9, but the dry weight of the strain is the smallest when the pH is 9, and the dry weight of the strain reaches the maximum when the pH is 7, which indicates that the optimal cultivation pH of Pb-9 is 7, and the strain is suitable for growth in a weak acid environment.

### 3.5. Analysis of the Adsorption Effect of Strain Pb-9 on Pb^2+^

**Figure 5:**
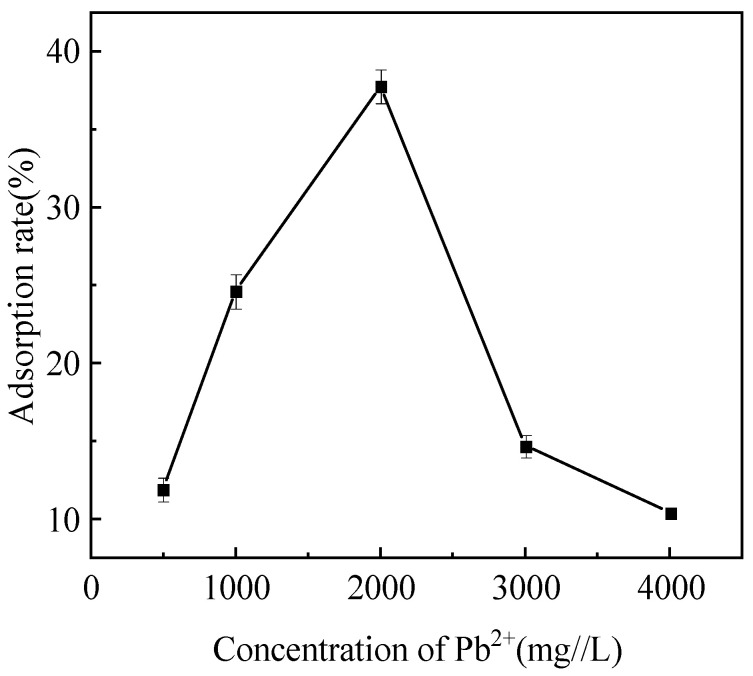
Adsorption rate of Pb^2+^ by strain Pb-9 at different Pb^2+^ concentrations.

The adsorption rate of Pb^2+^ by strain Pb-9 at different Pb^2+^ concentrations is shown in Figure 5. The results showed that the adsorption rate of lead ions gradually increased when the concentration of lead ions increased from 500 mg/L to 2000 mg/L. When the lead concentration was 2000 mg/L, the adsorption rate reached 37.75%. When the concentration of lead ions was more than 2000 mg/L, the adsorption rate started decreasing, which indicated that strain Pb-9 had a good adsorption effect on Pb^2+^ at a lead concentration of 2000 mg/L.

### 3.6. Scanning Electron Microscope Observation

**Figure 6:**
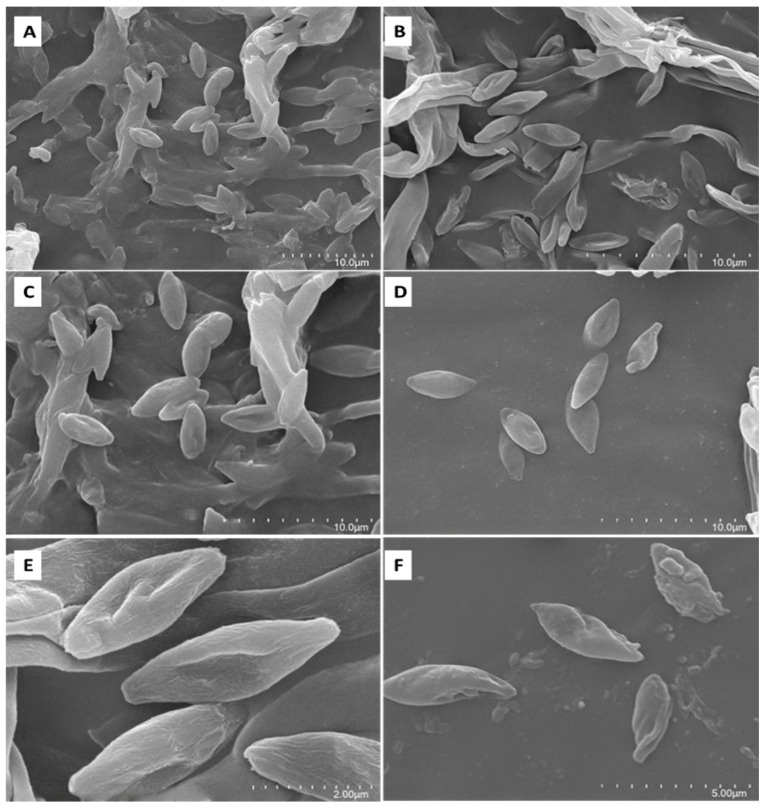
SEM micrographs of strain Pb-9 treated with different concentrations of Pb^2+^. Note: microstructure of mycelia: (**A**): CK; (**B**): 3000 mg/kg; microstructure of spores (**C**): CK; (**D**): 2000 mg/kg; (**E**): 3000 mg/kg; (**F**): 4000 mg/kg; (**A**) magnification times 3000×; (**B**) magnification times 5500×; (**C**,**D**) magnification times 5000×; (**E**) magnification times 20,000×; (**F**) magnification times 10,000×.

The mycelium and spore morphology of the fungus may have changed significantly after contact with toxic metals. The mycelium and spore morphology of the fungus Pb-9 in different Pb^2+^ concentration media were observed by scanning electron microscopy, as shown in Figure 6, the mycelium in the CK group was full, with a smooth surface, and the spore morphology was full, with fusiform and rounded spores (Figure 6A,C). However, with the increase in Pb^2+^ concentration, the mycelium and spore morphology underwent severe crumpling, twisting, and concavity (Figure 6B,D–F). When Pb^2+^ was 2000 mg/kg, the spore morphology did not change much compared with the CK group, the spore morphology was slightly wrinkled, and the ends of the spores were not rounded (Figure 6D). When Pb^2+^ was 3000 mg/kg, the surface of the spores of Pb-9 showed obvious wrinkles, the spore surface was not smooth, and the middle part was concave (Figure 6E). Finally, when Pb^2+^ was 4000 mg/kg, the spores of Pb-9 had been seriously twisted and deformed and were almost hammerless, the spore surface was uneven, and on the surface of some spores, there were small blocky bumps with blocky material attached to the spore surface (Figure 6F).

### 3.7. X-Ray Diffraction Analysis

**Figure 7:**
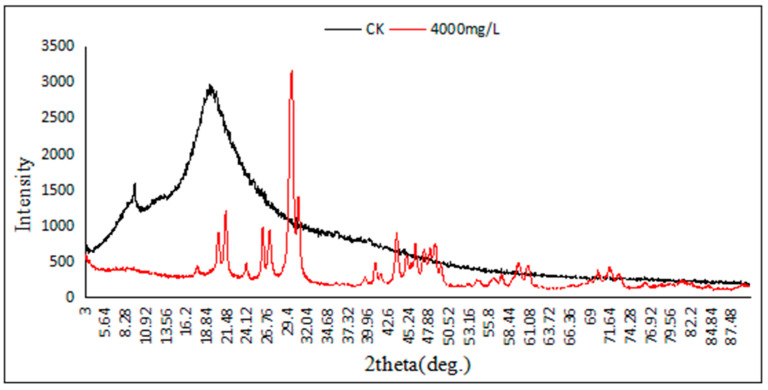
X-ray diffractogram of strain Pb-9 treated with different concentrations of Pb^2+^.

The XRD patterns of Pb-9 grown under 4000 mg/L and 0 mg/L (CK) Pb(NO_3_)_2_ treatments were analyzed using an X-ray diffractometer, respectively, and the results are shown in Figure 7. The XRD patterns of Pb-9 grown under 4000 mg/L and CK treatments were significantly different and analyzed using Jade 6.0 software. Swift analysis of the spectrum from a sample of 4000 mg/L allows us to assume that the formation of a crystalline substance—a mineral of natural origin—occurred. The peaks are very narrow and typical for good ordering. It was found that the presence of lead hydroxyl apatite (hydroxylpyromorphite, Pb₅(PO₄)₃OH) was based on the proximity of peak positions [14]. After a physical phase search, it was found that the substances corresponding to the characteristic peaks of Pb-9 under 4000 mg/L treatment all contained the Pb element, among which the characteristic peaks at diffraction angles of 30.16°–30.3° were the highest, while there were no obvious characteristic peaks of Pb-9 under CK treatment, so it can be inferred that the Pb element in Pb-9 mainly came from exogenous Pb(NO_3_)_2_ solution, indicating that this strain has a strong adsorption capacity for exogenous Pb^2+^.

### 3.8. Infrared Spectral Analysis of Pb-9 Strain Under Pb^2+^ Treatment

**Figure 8:**
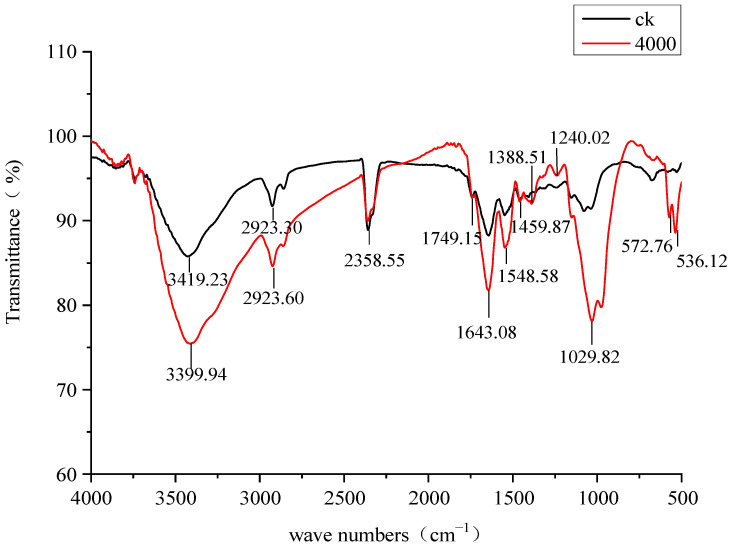
Infrared analysis of strain Pb-9 treated with different concentrations of Pd^2+^.

Figure 8 shows the IR absorption spectra of strain Pb-9 after 5 d of incubation with 4000 mg/L Pb^2+^ (treatment group) and without added Pb^2+^ (CK group), from which functional groups that may be involved in biosorption can be inferred. The treated and control groups showed characteristic absorption in the following spectral regions: in the 3700–3100 cm^−1^ interval, the maximum absorption of the bacteria in the control and treated groups was at 3419.23 cm^−1^ and 3399.94 cm^−1^, respectively, which is the vibrational absorption region of the O-H and N-H bond stretching. Among them, the stretching vibrations of the O-H groups make a larger contribution to the absorption band contours than the stretching vibrations of the N-H groups. The strong and broad absorption peaks at this location are the most significant among all absorption bands. In the 3000–2700 cm^−1^ interval, the maximum absorption of the bacteria in the control and treated groups was weakly to moderately strong at 2923.30 cm^−1^ and 2923.60 cm^−1^, respectively, and so was the C-H stretching vibrational absorption band on saturated carbon, which reflects information from the hydrophilic lipid molecules of fatty acids, various membranes, and cell wall components. In the interval from 1690 to 1500 cm^−1^, the maximum absorption of the bacteria in the control and treated groups was 1643.08 cm^−1^ and 1548.58 cm^−1^. The 1643 cm^−1^ peak combines the two most significant contributions: from the protein component (the Amide I peak, mainly the C=O stretching vibration with a smaller contribution from the NH_2_ deformation vibration) and water deformation vibrations (δ(H_2_O)). The peak of 1549 cm^−1^ belongs to the peak of the protein component—the peak of Amide II (predominantly NH_2_ deformation vibration). The 1750–1745 cm^−1^ interval is the C=O stretching vibration region of α-haloketone; there are several characteristic peaks in the 1460–1380 interval: the 1459.87 cm^−1^ peak is caused by the asymmetric vibration of CH_3_, the 1388.51 cm^−1^ peak is caused by the out-of-plane rocking of CH_2_ and the C-C-H variable angle vibration, and the 1240 cm^−1^ peak is the C-C stretching vibration. The 1240 cm^−1^ peak is caused by the C-C stretching vibration [15].

Both Pb^2+^ treated and CK groups had a strong absorption peak at 1029.82 cm^−1^, the stretching vibration of the C-N of the aliphatic amine group; the stretching vibration interval of the C-O bond in ester components and sugar rings was generally at 1360~1020 cm^−1^. Among them, the absorption intensity in this interval was significantly higher in the treatment group than in the control group, and these functional groups may be closely related to the adsorption of Pb^2+^ by the bacterium. Combined with the results obtained by the XRD method, the contribution of the inorganic component cannot be excluded either. The inorganic component explains the presence of narrow and atypical organic matter peaks doublet in the fingerprint region at 572.76 and 536.12 cm^−1^.

In conclusion, the infrared spectra of the treatment group and CK were the same, but there was a large difference in absorption intensity at 1029.82 cm^−1^, 3419.23 cm^−1^, and 3399.94 cm^−1^, which indicated that the bacterium might have participated in ester, fatty acid, and polysaccharide groups in the adsorption of Pb^2+^. The increase in absorption for the lead-containing sample occurs in the region of about 1000 cm^−1^, where the absorption bands of the inorganic substance (lead hydroxyl apatite) and the absorption bands in C-O bonds make their contribution [16].

## 4. Discussion

In lead-contaminated environments, certain taxa of microorganisms acquire resistance to lead toxicity due to chronic lead stress. Lihong Zhang [17] isolated two strains of fungi identified as *Sarocladium* sp. *M2* and *Sarocladium* sp. *M6* from coal mine soil. It was found that these two strains of fungi showed high resistance to cadmium, with 57.11 ± 4.45% removal of Cd(II) by M2 and 48.35 ± 1.44% removal of Cd(II) by M6 at an initial Cd(II) concentration of 200 mg/L. Văcar [18] found that *Cladosporium* sp., *Didymella glomerata*, *Fusarium oxysporum*, *Costaricphoma costaricensis*, and *kiliense Sarocladium* strains had high minimum inhibitory concentration values for Hg, Pb, Cu, Zn, and Cd. The biosorption of mercury by these highly mercury-resistant species ranged from 33.8 to 54.9 mg/g dry weight, with removal rates of 47 to 97%. In this paper, strain Pb-9 was also identified as *Sarocladium* by molecular biology, so it was judged that this strain could be used as a potential dominant bacterium for the bioremediation of heavy metal-contaminated soil.

Liang [19] reported that Aspergillusniger and Paecilomyces grown in a medium containing Pb(NO_3_)_2_ could produce phosphatase, and inositol hexakisphosphate and gamma-trihydro-2-phosphate could be enzymatically reacted under the action of phosphatase, releasing inorganic phosphate and producing oxalic acid. From this, Pb(NO_3_)_2_ in solution can be generated into lead oxalate and lead chlorophosphate precipitates, and lead chlorophosphate can be further converted to lead oxalate. The formation of these insoluble or weakly soluble lead oxalates modifies the potential biotoxicity of the heavy metal. *Penicillium simplicissimum* can produce oxalic acid during its growth, and a variety of heavy metal ions can be formed into precipitates with oxalic acid, which decreases the ecotoxicity of the heavy metal ions in the environment [3]. With other genera of fungal strains that have been reported to show similar properties, the present strain Pb-9 originated from soil that has been subjected to long-term Pb stress, and it was able to have high resistance to the heavy metal Pb. It can be hypothesized that this genus (*Sarocladium*) may also produce some secondary metabolites capable of chelating or reducing the ecotoxicity of heavy metals during their growth and metabolism. Therefore, strain Pb-9 is highly tolerant to Pb, and the specific adsorption mechanism of Pb^2+^ has to be studied in depth.

The trend in the adsorption rate of Pb^2+^ by strain Pb-9 at different Pb^2+^ concentrations can be explained by the fact that for a given mass of biomass, there are only a limited number of adsorption sites available, and these biomasses cannot accommodate the elevated concentration of Pb^2+^ in solution [20]. The reduced adsorption efficiency of Pb-9 for Pb^2+^ ions at high Pb^2+^ concentrations may be due to the inhibition of strain growth by high Pb^2+^ concentrations or due to the limited number of binding sites in the cell wall. Another study showed that when the concentration of Pb^2+^ ions reaches a certain level, the adsorption capacity tends to saturate, thus leading to a decrease in the adsorption rate [21].

The adsorption mechanisms of metal ions by the fungus are mainly: cell surface adsorption or complexation, intracellular enrichment, and exocytosis. Using scanning electron microscope observation, it was seen that the spore morphology of Pb-9 was wrinkled, deformed, and twisted under the treatment of a higher concentration of Pb^2+^, and some flakes with irregular morphology were attached to the surface of Pb-9 spores under the treatment of higher concentration. The results of XRD analysis showed that the Pb element was contained in Pb-9 under the treatment of Pb(NO_3_)_2_, while it was not detected in CK, which indicated that the Pb-9 Pb element in the strain comes from exogenously added Pb(NO_3_)_2_ solution, which can be inferred that Pb-9 has a strong adsorption effect on Pb(NO_3_)_2_.

Metal ions can undergo precipitation and redox reactions with biomolecules on the surface of microorganisms, and Wang Rong [22] reported the deposition of Pb^2+^ and Cd^2+^ inside and outside of the cells of resistant strains, whose functional groups on the cell wall -NH2, -OH, -COOH, -PO4 are mainly involved in the deposition and redox reactions of heavy metals, and there are also some literature reports that microorganisms produce ester and polysaccharide groups under heavy metal stress, which can chelate heavy metal ions and thus reduce the ecotoxicity of heavy metal ions to the environment [23]. Infrared analysis in this experiment also showed that a variety of functional groups in the Pb-9 strain, especially -OH, -N-H, C-O, C-C, -NO_2_, ester, and polysaccharide groups showed high activity after heavy metal Pb^2+^ treatment. However, it is impossible to ignore the possible contribution of the inorganic hydroxyapatite lead.

The strain Pb-9 screened in this study has high resistance and adsorption capacity to Pb, showing that it has a good prospect for development and application, and the follow-up research work can focus on its anti-Pb mechanism and improve its adsorption efficiency for in-depth investigation. Strain Pb-9 is expected to be developed and cultivated into a potentially advantageous strain in the bioremediation of soil heavy metal pollution.

## 5. Conclusions

In this experiment, a strain of Pb-resistant fungus (No. Pb-9) isolated from heavy metal-contaminated soil was investigated. The molecular biology method identified the strain (Pb-9) as Sarocladium, and scanning electron microscopy, infrared spectroscopy, and X-ray analysis were carried out to compare and analyze the changes in the micro-morphology of the strain and its adsorption of Pb^2+^ in different Pb^2+^ concentration treatments. The changes in the micro-morphology of the strain and its functional groups after Pb^2+^ adsorption were comparatively analyzed, and the resistance and adsorption mechanism of the strain to Pb^2+^ were preliminarily revealed. The study of this strain in this paper provides a fundamental basis for its use as a potential dominant strain for the bioremediation of heavy metal-contaminated soil.

## Data Availability

The sequence data of the strains identified in the article have been uploaded on the NCBI website under the accession number MK372219.
